# Prediction of steady flows passing fixed cylinders using deep learning

**DOI:** 10.1038/s41598-021-03651-8

**Published:** 2022-01-10

**Authors:** Hiroto Ozaki, Takeshi Aoyagi

**Affiliations:** Research Center for Computational Design of Advanced Functional Materials, National Institute of Advanced Industrial Science and Technology, Central 2, 1-1-1, Umezono, Tsukuba, Ibaraki 305-8568 Japan

**Keywords:** Computational science, Computational methods, Fluid dynamics

## Abstract

Considerable attention has been given to deep-learning and machine-learning techniques in an effort to reduce the computational cost of computational fluid dynamics simulation. The present paper addresses the prediction of steady flows passing many fixed cylinders using a deep-learning model and investigates the accuracy of the predicted velocity field. The deep-learning model outputs the *x*- and *y*-components of the flow velocity field when the cylinder arrangement is input. The accuracy of the predicted velocity field is investigated, focusing on the velocity profile of the fluid flow and the fluid force acting on the cylinders. The present model accurately predicts the flow when the number of cylinders is equal to or close to that set in the training dataset. The extrapolation of the prediction to a smaller number of cylinders results in error, which can be interpreted as internal friction of the fluid. The results of the fluid force acting on the cylinders suggest that the present deep-learning model has good generalization performance for systems with a larger number of cylinders.

## Introduction

Computational fluid dynamics (CFD) simulation^1^ has been hugely successful in the field of engineering. The scope of its application has broadened as a result of improved simulation techniques. One example of a simulation technique that has been improved in recent years is the modeling of fluid–structure interactions^2, 3^. Fluid–structure interactions are widely analyzed in various fields; for example, in studying heart–blood interactions^4^, wind-induced effects on buildings^5^, and the flight of insects with flexible wings^6^. Meanwhile, the interaction between a fluid and solid has been studied in investigations of microscopic physical phenomena in the fields of materials science and chemical engineering^7–11^. Simulations in these fields deal with complex geometries, such as those of porous media^7–10^, and multiphase flows, such as fluid flows containing a large number of particles^11^. Even with the development of computer and simulation technologies, the well-recognized problem of the computational cost is yet to be solved because the complexity of the simulations is increasing.

In recent years, deep-learning^12^ and machine-learning^13^ techniques have been applied to engineering problems with great success: medical image processing^14^, designs of materials^15^, and remaining useful life estimations^16, 17^. The techniques are also attracting attention from the viewpoint of reducing the computational cost of CFD simulations^18–22^. Guo et al. proposed a model for the real-time prediction of a steady flow passing an object based on convolutional neural networks^18^. In their study, the velocity field around an object was estimated efficiently using convolutional neural networks. Hennigh later improved the network model of Guo et al. and applied it to the shape optimization of an airfoil and heat sink^20^. With the goal of real-time CFD simulation, Umetani and Bickel presented a technique of predicting the drag coefficient, the pressure acting on the surface of an object, and the velocity field around an object adopting a Gaussian process^21^. Liang et al. constructed deep neural networks to predict the steady-state distributions of the pressure and flow velocity inside the thoracic aorta^22^. Their study showed that deep neural networks have the potential to be used in place of CFD simulation for the steady-state hemodynamic analysis of human blood vessels. The successes of the deep-learning and machine-learning techniques will lead to the reduction of the computational cost of simulations that deal with fluids and solids in materials science and chemical engineering.

The present research applies a deep-learning technique to the smoothed profile method (SPM)^23^, a simulation method for multiphase flows with solid objects. The present research focuses on the flow around many cylinders and consists of the following two studies. The first of the two studies in the present research predicts a steady flow passing fixed cylinders, which is reported on in the present paper. In this study, all cylinders are considered to be immobile and spatially fixed. The fluid flow is induced by a boundary condition. Based on the work by Hennigh^20^, this study constructs a deep-learning model having a U-Net-like architecture^24^. The U-Net is typically applied in image segmentation problems, but as Hennigh did, the present study applies the model to a physics problem. The accuracy of the predicted velocity field, in terms of the velocity profile of the fluid flow and the fluid force acting on the cylinders, is investigated. The second study, which will be reported on in an adjoining paper, addresses the prediction of the steady flow induced by moving cylinders. In the second study, another deep-learning model that treats the velocity of the cylinders is constructed by extending the present model.

The remainder of this paper is organized as follows. Section “Simulation method” describes details of the CFD simulation method, namely the SPM, used in preparing the dataset. Section “Problem setup” presents the problem addressed in this study, namely a steady flow passing many fixed cylinders. Section “Deep-learning model” presents the constructed deep-learning model. Section “Results and discussion” presents the results and a discussion, where the accuracy of the predicted velocity field is examined. Section “Conclusion” presents conclusions drawn from the results of the study.

## Simulation method

This section presents details of the CFD simulation conducted to prepare the dataset.

The study deals with two-dimensional flows around cylinders. A number of methods have been proposed to deal with fluid flow around solid objects, and the SPM^23^ is used in the present study because the description of the solid objects (i.e., cylinders in this study) in the SPM is tractable as input data for deep learning, as shown in the next section. In the SPM, the interface between the fluid and a solid object (i.e., cylinder) is described by a continuous smoothed function that takes a value of 1 inside the solid and 0 outside the solid. This allows the direct and efficient numerical simulation of the fluid with the solid objects on a fixed Cartesian grid without a remeshing process. The details of the SPM have been presented in previous reports^23, 25, 26^, and only an overview of the method is given here.

First, the smoothed function representing the cylinders is described. The smoothed function of the *i*th cylinder at position ${\mathbf {R}}_{i}$ is defined as a function of the spatial coordinates ${\mathbf {x}}$:

1 $$\begin{aligned} \phi _{i}({\mathbf {x}})&= g(|{\mathbf {x}} - {\mathbf {R}}_{i}|), \end{aligned}$$

2 $$\begin{aligned} g(x)&= \frac{h[(A+\xi /2)-x]}{h[(A+\xi /2)-x] + h[x - (A -\xi /2)]}, \end{aligned}$$

3 $$\begin{aligned} h(x)&= {\left\{ \begin{array}{ll} \exp (-d^2/x^2) &{} { x} \ge 0, \\ 0 &{} { x} < 0, \end{array}\right. } \end{aligned}$$

where *A* is the radius of the cylinder, $\xi$ is the interfacial thickness, and *d* is the lattice spacing. Accordingly, when there are *N* cylinders in the system, the smoothed function for all the cylinders is

4 $$\begin{aligned} \phi = \sum _{i}^{N} \phi _{i}. \end{aligned}$$

Using the function $\phi$, the total velocity field ${\mathbf {u}}$ is expressed as

5 $$\begin{aligned} {\mathbf {u}} = \phi {\mathbf {u}}_{\mathrm {c}} + (1-\phi ) {\mathbf {u}}_{\mathrm {f}}, \end{aligned}$$

where ${\mathbf {u}}_{\mathrm {c}}$ and ${\mathbf {u}}_{\mathrm {f}}$ respectively represent the velocity fields of the cylinder region and fluid region. It is noted that the velocity field of the cylinder region ${\mathbf {u}}_{\mathrm {c}}$ takes the value 0 in the present study because this study considers fixed cylinders.

The time development of the total velocity field ${\mathbf {u}}$ is given by the Navier–Stokes (NS) equation:

6 $$\begin{aligned} \frac{\partial {\mathbf {u}}}{\partial t} + ({\mathbf {u}} \cdot {\mathbf {\nabla }}) {\mathbf {u}}&= - \frac{1}{\rho } {\mathbf {\nabla }} p + \nu \nabla ^2 {\mathbf {u}} + \phi {\mathbf {f}}_{\mathrm {c}}, \end{aligned}$$

where $\rho$ is the density of the fluid, *p* is the pressure, and $\nu$ is the kinematic viscosity. The pressure *p* is determined so as to retain the incompressibility condition: ${\mathbf {\nabla }} \cdot {\mathbf {u}} = 0$. The term $\phi {\mathbf {f}}_{\mathrm {c}}$ represents the body force acting on the fluid due to the existence of the cylinders. In the SPM, the force is determined so as to make the velocity field of the cylinder region consistent with the cylinder velocity. (The details are given later in Eq. 11.) It is noted that the SPM imposes a no-slip boundary condition on the fluid–structure interface because the tangential velocity difference is reduced by the viscous stress even on the interface^23^. Meanwhile, the hydrodynamic force ${\mathbf {F}}_{i}^{\mathrm {H}}$ acting on the *i*th cylinder is obtained as

7 $$\begin{aligned} {\mathbf {F}}_{i}^{\mathrm {H}}&= - \int _{V_{\mathrm {c}}} \rho \phi _{i} {\mathbf {f}}_{\mathrm {c}} d {\mathbf {x}}, \end{aligned}$$

where $\int _{V_{\mathrm {c}}}$ represents the volume integration of the cylinder region.

In the present simulation, the NS equation (6) is solved adopting a fractional step scheme on a semi-staggered grid^27^ as follows. First, the evolution equation that removes the pressure and body force terms is considered:

8 $$\begin{aligned} \frac{\tilde{{\mathbf {u}}}^{*} - {\mathbf {u}}^{n}}{\Delta \tau } + ({\mathbf {u}}^{n} \cdot {\mathbf {\nabla }}) {\mathbf {u}}^{n} - \nu \nabla ^2 {\mathbf {u}}^{n} = 0, \end{aligned}$$

where the forward difference is applied in the time discretization. The term $\Delta \tau$ is the pseudo-time increment used in obtaining the steady-state values. The velocity $\tilde{{\mathbf {u}}}^{*}$ is the provisional total velocity, and ${\mathbf {u}}^{n}$ is the total velocity obtained in step *n*. Next, the pressure term is considered. The term is determined so as to make the provisional total velocity divergence free by solving the Poisson equation:

9 $$\begin{aligned} \nabla ^2 p^{*}&= \frac{\rho }{\Delta \tau } ({\mathbf {\nabla }}\cdot \tilde{{\mathbf {u}}}^{*}). \end{aligned}$$

In the present simulation, the Poisson equation is solved adopting the Bi-CGSTAB method^28^. Considering the pressure term, the divergence-free provisional velocity $\tilde{{\mathbf {u}}}^{n+1}$ is obtained as

10 $$\begin{aligned} \tilde{{\mathbf {u}}}^{n+1}&= \tilde{{\mathbf {u}}}^{*} - \frac{\Delta \tau }{\rho } {\mathbf {\nabla }} p^{*}. \end{aligned}$$

The term for the body force acting on the fluid is then considered. The term is determined so as to make the fluid velocity in the cylinder region consistent with the cylinder velocity^26^:

11 $$\begin{aligned} \phi {\mathbf {f}}_{\mathrm {c}} \Delta \tau = \phi ({\mathbf {u}}_{\mathrm {c}} - \tilde{{\mathbf {u}}}^{n+1}). \end{aligned}$$

Eventually, the velocity field in step $n+1$ is obtained as

12 $$\begin{aligned} {\mathbf {u}}^{n+1} = \tilde{{\mathbf {u}}}^{n+1} + \phi {\mathbf {f}}_{\mathrm {c}} \Delta \tau - \frac{\Delta \tau }{\rho } {\mathbf {\nabla }} \tilde{p}^{n+1}, \end{aligned}$$

where $\tilde{p}^{n+1}$ is an additional pressure term included so as to make the velocity ${\mathbf {u}}^{n+1}$ divergence free.

In the present study, the steady flow is obtained by fixing the cylinder position and solving the NS equation iteratively. The iteration ends when the unsteady effect becomes negligible in terms of the total velocity field; in other words, the total velocity field ${\mathbf {u}}^{n+1}$ is considered the same as that in the previous step ${\mathbf {u}}^{n}$. Here, the criterion with which to interrupt the iteration is set as

13 $$\begin{aligned} \mathrm {err}^{n+1} \equiv \frac{\sum _{i,j}\sqrt{[u^{n+1}(i,j) - u^{n}(i,j)]^2 + [v^{n+1}(i,j) - v^{n}(i,j)]^2}}{\sum _{i,j}\sqrt{u^{n+1}(i,j)^2 + v^{n+1}(i,j)^2}} < {1\times 10^{-9}}, \end{aligned}$$

where $u^{n+1}(i,j)$ and $v^{n+1}(i,j)$ ($u^{n}(i,j)$ and $v^{n}(i,j)$) are respectively the *x*- and *y*-components of the total velocity field of the present step (the previous step) at the calculation point (*i*, *j*). It is noted that the obtained steady flow slightly depends on the pseudo-time increment $\Delta \tau$. The pseudo-time increment $\Delta \tau$ is therefore fixed to a single value throughout the present study as described in the next section.

## Problem setup

**Figure 1:**
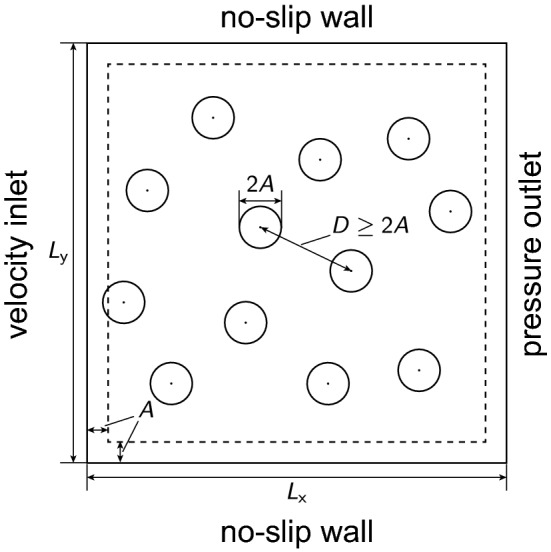
Schematic diagram of the analysis domain defined in the present study. The circles represent cylinders and the dashed square represents the region in which the center of each cylinder lies.

This section describes in detail the addressed problem, namely a steady flow passing fixed cylinders. Figure 1 is a schematic diagram of the analysis domain used in solving this problem. A velocity inlet boundary condition and a pressure outlet boundary condition are imposed on the left and right walls of the analysis domain, respectively. A no-slip boundary condition is imposed on the top and bottom walls. As a result, the flow direction is from left to right. Cylinders are randomly placed in a way that they do not overlap each other or boundaries in the analysis domain. (The cylinders are represented as circles in Fig. 1.) The interaction between the cylinders and fluid is described using the SPM (see “Simulation method”), and a no-slip boundary condition is imposed on the interfaces of the cylinders.

The training data are prepared in the CFD simulation described in “Simulation method”. The parameters of the simulation are set as follows. The units of length and time are given by the lattice spacing *d* and $d^2 / \nu$, respectively, where $d = 1$ and $\nu = 1$. The density of the fluid is set as $\rho = 1$. The system size is $L_{\mathrm {x}} = L_{\mathrm {y}} = 127$. The number of calculation points is then $128 \times 128$. The cylinder radius is $A = 4$, and the interface thickness is $\xi = 2$. The settings for *A* and $\xi$ are adopted from the previous study^25^, where the rheology of colloidal dispersions is successfully described. At the velocity inlet boundary (i.e., left wall), a parabolic flow velocity profile (i.e., a plane Poiseuille flow^29^) is set; that is, the *x*-component of the velocity at the boundary is given by

14 $$\begin{aligned} u(y) = 6 {\bar{u}} \frac{y}{L_{\mathrm {y}}} \left( 1 - \frac{y}{L_{\mathrm {y}}}\right) , \end{aligned}$$

where ${\bar{u}}$ is the mean flow velocity and is set as ${\bar{u}} = 0.1$ in the present study. The *y*-component of the velocity at the boundary is set as $v = 0$. The pseudo-time increment is set as $\Delta \tau = 0.1$. The cylinders are placed so as not to overlap other cylinders or the boundary of the analysis domain. Here, the distance between the centers of cylinders, which is denoted *D* in Fig. 1, is kept larger than or equal to the diameter of a cylinder 2*A*. The distances between the walls and the center of each cylinder are kept larger than or equal to the radius of the cylinder *A*. (The center of each cylinder lies within the dashed square.)

The flow obtained in the simulation is considered to be steady for the following reasons. First, in the present study, the Reynolds number based on the cylinder diameter is $\mathrm {Re}_{\mathrm {c}} \equiv 2 {\bar{u}} A / \nu = 0.8$, which is smaller than the typical Reynolds number ($\mathrm {Re}_{\mathrm {c}} \gtrsim 40$) where the unsteady flow is observed^1, 30^. Second, in the present simulation, temporal increases and decreases in the fluid velocity were not observed, and the amount of change in a simulation step (defined in Eq. 13) converged.

## Deep-learning model

**Figure 2:**
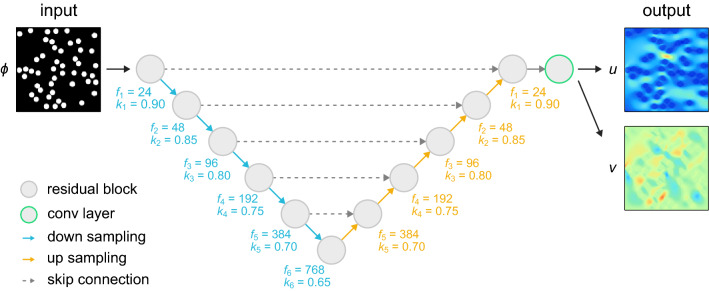
Schematic representation of the architecture of the deep-learning model. The input is a smoothed profile function $\phi$, and the outputs are the *x*- and *y*-components of the flow velocity (i.e., *u* and *v*). The filter size $f_{i}$ and keep probability $k_{i}$ of *i*th residual block are shown.

This section describes the architecture of the deep-learning model used in the present study. With the cylinder arrangement given as the input, the present model outputs the fluid velocity field. In the model, the cylinder arrangement is described by the smoothed profile function $\phi$ and treated as an array of values with the size of $128 \times 128$. (The detail is shown later.) The fluid velocity field is treated as an array of values with the size of $128 \times 128 \times 2$, since the velocity defined at each calculation point is a vector quantity and has two components (that is, *x*- and *y*-components). The present model is based on a model proposed by Hennigh^19, 20^. Hennigh’s model uses U-net with gated residual blocks^24, 31^, which allows for the efficient learning of global information of the boundary of structures. Several modifications to Hennigh’s methodology are made so that it is applicable to the present problem.

Figure 2 is a schematic representation of the network architecture used to solve the present problem. The present model is different from Hennigh’s model^19^ in the following three respects. The effectiveness of the modifications is investigated by additional studies. The result is shown in Supplementary Information.

### Input data

When using Hennigh’s model^19^, the information of the boundary of structures is input in a binary fashion; that is, the fluid and structure regions are respectively represented as 0 and 1. When using the present model, the information is input as the field of the smoothed profile function $\phi$. In the smoothed profile function, the fluid–structure interface is represented as values between 0 and 1, and the position of the interface is described in more detail than in the binary representation.

### Degrees of freedom

To predict the more complex flow, the present model has more degrees of freedom than Hennigh’s model. Compared with Hennigh’s model^19^, the number of down-sample and up-sample operations is increased. The filter size is also increased with reference to a wide residual network^32^. Figure 2 presents specific values of the filter size. Meanwhile, to train a model with high complexity, a different keep probability of the dropout layer is assigned to each residual block. The probability is set as 0.9 for the first block and decreases in steps of 0.05 for deeper blocks.

### Normalization technique

To realize stable training, batch normalization is applied before the activation function, which is not done in the case of Hennigh’s model. The activation function used in the present model is the concatenated exponential linear unit function^33, 34^, which is the same function used in Hennigh’s model.

A set of 24,000 samples (8000 samples for each of the systems having $N = 32$, 48, and 64 cylinders) was generated to train the model. Each sample contains the smoothed function $\phi$ and the *x*- and *y*-components of flow velocity, namely *u* and *v*. The data are augmented by adding a vertical flip of each original sample: for $\phi$ and *u*, the values at the calculation point (*i*, *j*) in the flipped sample are the same as those at the calculation point $(i, N_{\mathrm {y}} - j)$ of the original sample; for *v*, the value at the calculation point (*i*, *j*) in the flipped sample is the same as the sign-inverted value at the calculation point $(i, N_{\mathrm {y}} - j)$ of the original sample.

The present model is trained so as to minimize the loss function

15 $$\begin{aligned} \mathrm {loss} \equiv \frac{\sum _{i,j}\{[u^{\mathrm {pred}}(i,j) - u^{\mathrm {true}}(i,j)]^2 +[v^{\mathrm {pred}}(i,j) - v^{\mathrm {true}}(i,j)]^2\}}{2}, \end{aligned}$$

where $u^{\mathrm {pred}}(i,j)$ and $v^{\mathrm {pred}}(i,j)$ are respectively the *x*- and *y*-components of the predicted velocity at the calculation point (*i*, *j*). Similarly, $u^{\mathrm {true}}(i,j)$ and $v^{\mathrm {true}}(i,j)$ are respectively the *x*- and *y*-components of the true velocity at the calculation point (*i*, *j*). The mini-batch size and learning rate are respectively set to 8 and ${1\times 10^{-5}}$ throughout the learning process. The present study utilizes TensorFlow^35^ version 1.12.0 to create the deep-learning model and runs it on a single node of NVIDIA$^{\textregistered }$ Tesla$^{\textregistered }$ V100 GPU.

## Results and discussion

**Figure 3:**
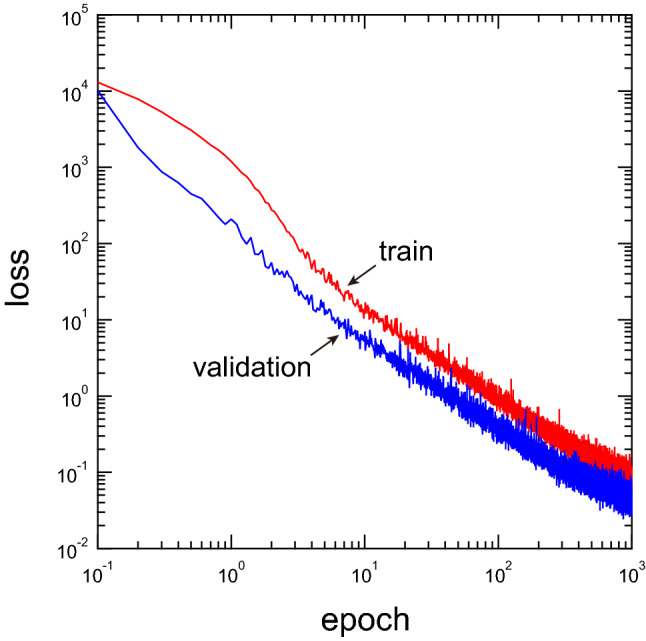
Training and validation losses per mini-batch as functions of the number of epochs (red: training loss, blue: validation loss).

Figure 3 shows the training and validation losses per mini-batch during the learning. The loss decreases with an increase in the number of epochs, and it is confirmed that the present model successfully learns the steady flow. No overfitting is observed during the learning process. During the learning, the validation loss tends to be lower than the training loss. This is due to the disabling of the dropout layers during the validation. When the keep probability of the dropout layer is set to 1 (i.e., the dropout layer is disabled throughout the learning and validation processes), the two losses take almost the same value. Hereinafter, the prediction made by the model at 1000 epochs is investigated.

**Figure 4:**
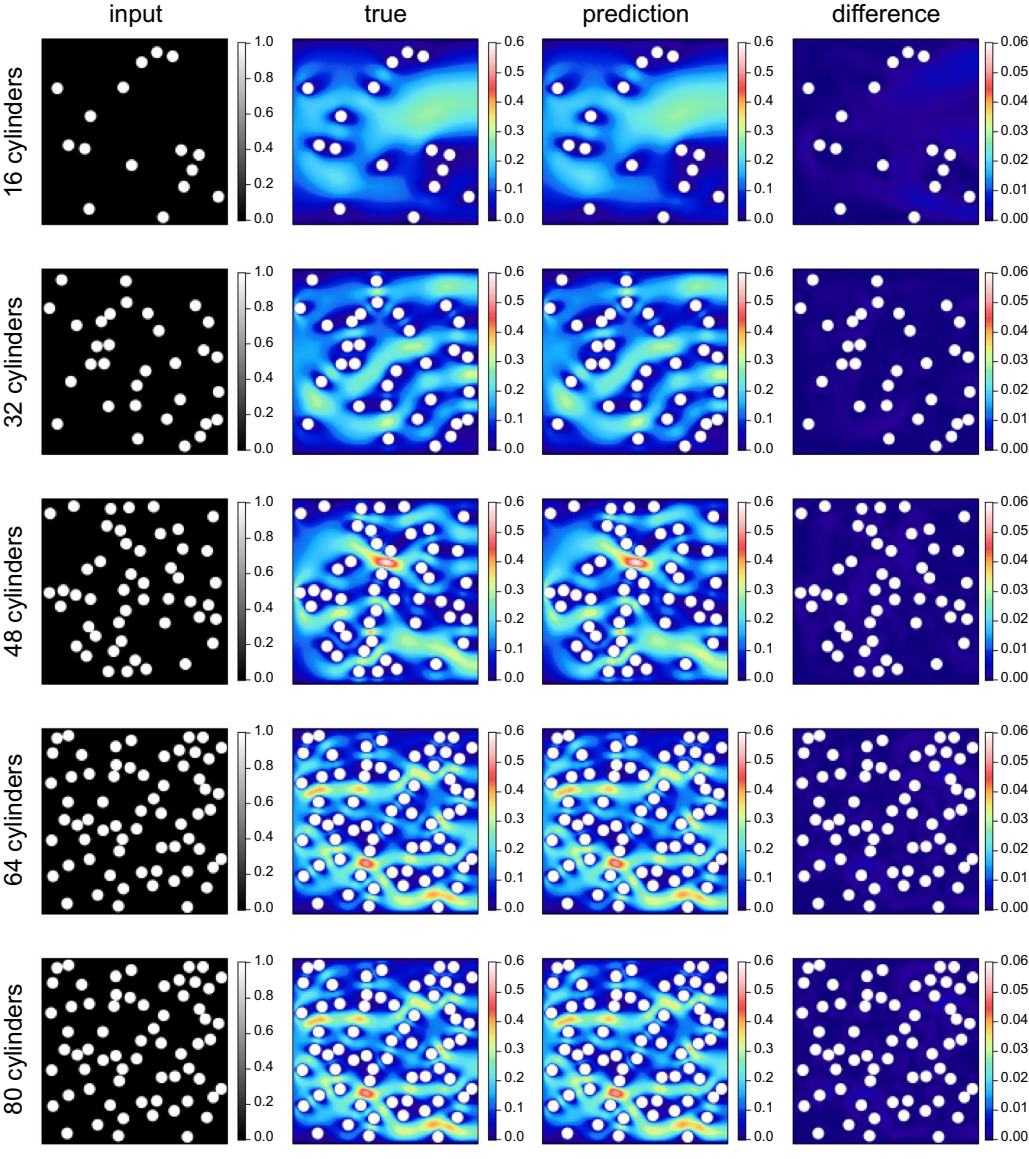
Comparison of the true and predicted velocity fields for unknown cylinder systems that are not learned in the training and validation datasets. The leftmost column shows the input data (the smoothed profile function $\phi$). The rest, from left to right, show the magnitude of the true velocity, predicted velocity, and difference between the true and predicted velocities. The rows from top to bottom respectively show the results for the systems with 16, 32, 48, 64, and 80 cylinders. To make the cylinder position easier to see, the velocity values defined in the cylinder domain are shown in white.

Next, the true and predicted velocity fields are compared for unknown cylinder systems that are not learned in the training and validation datasets (Fig. 4). In the figure, the columns from left to right show input data that represent the cylinder arrangement (i.e., the smoothed profile function $\phi$), the magnitude of the true velocity $\sqrt{\left[ u^{\mathrm {true}}(i,j)\right] ^2 + \left[ v^{\mathrm {true}}(i,j)\right] ^2}$, the magnitude of the predicted velocity $\sqrt{\left[ u^{\mathrm {pred}}(i,j)\right] ^2 + \left[ v^{\mathrm {pred}}(i,j)\right] ^2}$, and the magnitude of the difference between the true and predicted velocities $\sqrt{\left[ \Delta u(i,j) \right] ^2 + \left[ \Delta v(i,j) \right] ^2}$, where $\Delta u(i,j) \equiv u^{\mathrm {true}}(i,j) - u^{\mathrm {pred}}(i,j)$ and $\Delta v(i,j) \equiv v^{\mathrm {true}}(i,j) - v^{\mathrm {pred}}(i,j)$. In the comparison, the number of cylinders *N* is varied from 16 to 80 in intervals of 16. It is noted that the systems for which $N = 16$ and 80 have not been seen by the model during the training. For the “difference” field, the range of the color bar is set 10 times narrower than that for the “true” and “prediction” fields. In the results for all *N*, the predicted velocity fields agree well with the true velocity fields. It is, however, qualitatively seen from the results of the “difference” field that the prediction with small *N* has lower accuracy than that with large *N*. For small *N*, the deep-learning model predicts the velocity at the calculation points far from the cylinder interface. To achieve the prediction accurately, the model needs to learn global information during the training process.

**Figure 5:**
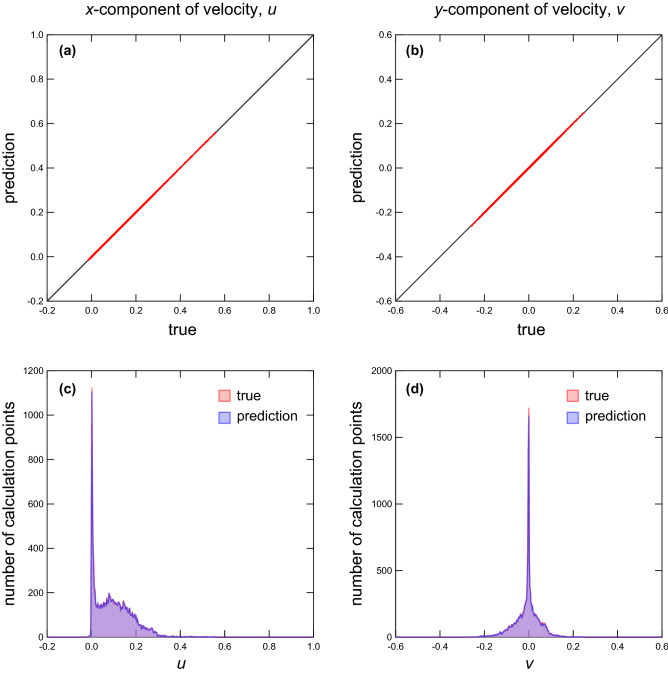
Scatter plots (**a**,**b**) and histograms (c and d) of the true and predicted flow velocities of all calculation points. The number of cylinders is set at $N = 48$. (**a**) Scatter plot of true and predicted *x*-components of the flow velocity *u*. (**b**) Scatter plot of true and predicted *y*-components of the flow velocity *v*. (**c**) Histograms of true (red) and predicted (blue) *x*-components of the flow velocity *u*. (d) Histograms of true (red) and predicted (blue) *y*-components of the flow velocity *v*.

The flow velocity is investigated at each calculation point to examine the predicted velocity field in detail. Figure 5 shows scatter plots (a,b) and histograms (c,d) of the true and predicted velocity values at all calculation points ($N = 48$). The results show that the predicted velocities agree with the true velocities, but there is a slight difference in the height of the peaks in the histograms. The peaks are observed at $u = 0$ and $v = 0$, and they can be attributed to the boundary condition. The result therefore implies that the predicted velocity values at the boundary fluctuate.

**Figure 6:**
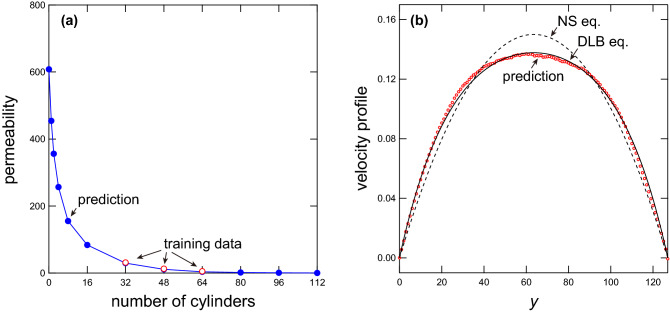
(**a**) Permeability *k* as a function of the number of cylinders. Red open and blue closed symbols indicate the permeabilities obtained from the training data and prediction, respectively. (**b**) Velocity profile predicted using the present model when the cylinders are not present (red symbols). The solid line indicates the velocity profile obtained using the DLB equation with the permeability $k \simeq 608$ (see Eq. 17). The dashed line indicates the velocity profile obtained using the NS equation (see Eq. 14).

The predicted flow field is next investigated from a physical perspective. A fluid flowing past many fixed cylinders, which is the problem addressed in this study, can be considered as a fluid flowing through a complex geometry such as the geometry of a porous medium. It is therefore expected that the averaged fluid flow passing randomly placed cylinders can be described by the Darcy–Lapwood–Brinkman (DLB) equation, which treats the bulk velocity of a flow passing through a porous medium by introducing a Darcy friction term^36, 37^. The DLB equation is written as

16 $$\begin{aligned} \frac{\partial {\hat{{\mathbf {u}}}}}{\partial t} + ({\hat{{\mathbf {u}}}} \cdot {\mathbf {\nabla }}) {\hat{{\mathbf {u}}}}&= - \frac{1}{\rho } {\mathbf {\nabla }} p - \frac{\nu }{k} {\hat{{\mathbf {u}}}} + \nu _{\mathrm {eff}} \nabla ^2 {\hat{{\mathbf {u}}}}, \end{aligned}$$

where ${\hat{{\mathbf {u}}}}$ is the ensemble-averaged velocity within the porous medium, *k* is the permeability, and $\nu _{\mathrm {eff}}$ is the kinematic effective viscosity of the fluid in the medium. When *k* is infinite, the equation has the same form as the NS equation. Using the DLB equation, the velocity profile between parallel plates is obtained as

17 $$\begin{aligned} \hat{u}(y) = {\bar{u}} \dfrac{2 L'_{\mathrm {y}} \sinh \left( y'\right) \sinh \left( L'_{\mathrm {y}} - y' \right) }{L'_{\mathrm {y}} \cosh \left( L'_{\mathrm {y}}\right) - \sinh \left( L'_{\mathrm {y}}\right) }, \end{aligned}$$

where $L'_{\mathrm {y}} \equiv L_{\mathrm {y}} / 2 \sqrt{k}$ and $y' \equiv y / 2 \sqrt{k}$. In the derivation, $\nu = \nu _{\mathrm {eff}}$ is assumed. By fitting the average velocity profile of the predicted flow with Eq. (17), the permeability *k* is obtained for each number of cylinders *N* and the dependence of *k* on *N* is investigated. The results are shown in Fig. 6(a) (blue closed symbols). Here, the velocity profiles to be fitted with Eq. (17) are obtained by averaging the 10, 000 different velocity profiles on the outlet boundary (i.e., the velocity value at $x = L_{\mathrm {x}}$) for each *N*. In the figure, the permeability *k* obtained in the same manner from the training data is shown with red open symbols. The permeability obtained from the predicted velocity profiles agrees well with that obtained from the training data. Meanwhile, the value of *k* obtained from the predicted velocity profiles increases rapidly as *N* decreases. Theoretically, the permeability *k* should diverge to infinity at $N = 0$. In the present study, however, the predicted velocity profile at $N = 0$ is well described by the DLB equation with $k \simeq 608$ (Fig. 6b). This result indicates that, at $N = 0$, the resistance force is acting on the fluid in the form of internal friction, although the flow satisfies the incompressibility condition and preserves the mean flow velocity. It is considered that the present deep-learning model learns the flow field such that it reproduces the physics of the DLB equation, and it has an error determined by the internal friction when *N* is small. Given the range of *N* in the training dataset, the error found when *N* is small is probably due to the large extrapolation to lower values of *N*. The issue is not crucial when preparing the training data with appropriate cylinder numbers that match the conditions of the prediction.

**Figure 7:**
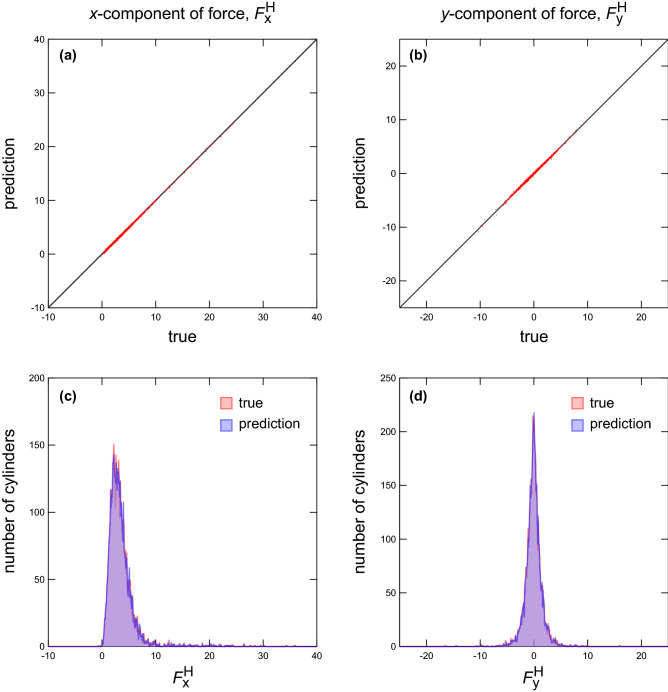
Scatter plots (**a**,**b**) and histograms (**c**,**d**) of the true and predicted forces acting on each cylinder. The number of cylinders is set at $N = 48$. The figure shows the gathered results of 100 patterns of the cylinder arrangement. (**a**) Scatter plot of the *x*-components of the true and predicted forces acting on each cylinder. (**b**) Scatter plot of the *y*-components of the true and predicted forces acting on each cylinder. (**c**) Histograms of *x*-components of the true (red) and predicted (blue) forces acting on each cylinder. (**d**) Histograms of *y*-components of the true (red) and predicted (blue) forces acting on each cylinder.

Lastly, the force acting on a cylinder is compared between the prediction and ground truth. The force acting on the *i*th cylinder ${\mathbf {F}}_{i}^{\mathrm {H}}$ is obtained as follows. First, for the obtained steady-state flow velocity field, the pseudo-time evolution is performed (see Eqs. 8–10). The body force acting on the fluid $\phi {\mathbf {f}}_{\mathrm {c}}$ (and $\phi _{i} {\mathbf {f}}_{\mathrm {c}}$) is then obtained from the velocity field using Eq. (11). The force ${\mathbf {F}}_{i}^{\mathrm {H}}$ is then obtained from Eq. (7). Hereinafter, the forces obtained from the true and predicted flow fields are respectively referred to as the “true force” and “predicted force”. Figure 7 presents scatter plots (a and b) and histograms (c and d) of the true and predicted forces ${\mathbf {F}}_{\mathrm {true}}^{\mathrm {H}}$ and ${\mathbf {F}}_{\mathrm {pred}}^{\mathrm {H}}$ ($N = 48$). (In the notation of the forces, the subscript letter *i* is omitted because it is no longer necessary to specify the cylinder number.) The figure shows the gathered results of 100 patterns of the cylinder arrangement. A comparison of the true and predicted forces acting on each cylinder confirms the accuracy of the predicted velocity field.

**Figure 8:**
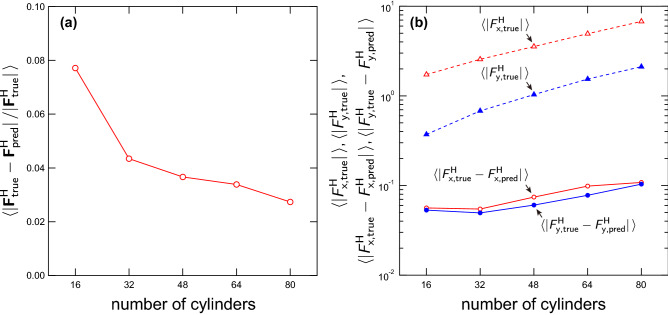
(**a**) Average relative error for the forces acting on cylinders $\langle |{{\mathbf {F}}^{\mathrm {H}}_{\mathrm {true}} - {\mathbf {F}}^{\mathrm {H}}_{\mathrm {pred}}}| / |{{\mathbf {F}}^{\mathrm {H}}_{\mathrm {true}}}| \rangle$ as a function of the cylinder number *N*. (**b**) Average absolute errors for the *x*- and *y*-components of the forces, $\langle |{F^{\mathrm {H}}_{\mathrm {x, true}} - F^{\mathrm {H}}_{\mathrm {x, pred}}}| \rangle$ (red open circles) and $\langle |{F^{\mathrm {H}}_{\mathrm {y, true}} - F^{\mathrm {H}}_{\mathrm {y, pred}}}| \rangle$ (blue closed circles), acting on cylinders as a function of the cylinder number *N*. The average values of *x*- and *y*-components of forces acting on cylinders obtained by numerical simulation, $\langle |{F^{\mathrm {H}}_{\mathrm {x, true}}}| \rangle$ and $\langle |{F^{\mathrm {H}}_{\mathrm {y, true}}}| \rangle$, are shown with red and blue triangles, respectively.

The average relative error for the forces acting on cylinders $\langle |{{\mathbf {F}}^{\mathrm {H}}_{\mathrm {true}} - {\mathbf {F}}^{\mathrm {H}}_{\mathrm {pred}}}| / |{{\mathbf {F}}^{\mathrm {H}}_{\mathrm {true}}}| \rangle$ (Fig. 8a) is investigated next. The results show that the error increases as the number of cylinders *N* decreases. To examine this result in detail, the average absolute errors for the *x*- and *y*-components of the forces $\langle |{F^{\mathrm {H}}_{\mathrm {x, true}} - F^{\mathrm {H}}_{\mathrm {x, pred}}}| \rangle$ and $\langle |{F^{\mathrm {H}}_{\mathrm {y, true}} - F^{\mathrm {H}}_{\mathrm {y, pred}}}| \rangle$ are also investigated (Fig. 8b, circles). The figure also shows the average values of *x*- and *y*-components of forces $\langle |{F^{\mathrm {H}}_{\mathrm {x, true}}}| \rangle$ and $\langle |{F^{\mathrm {H}}_{\mathrm {y, true}}}| \rangle$ obtained in the numerical simulation (triangles). Meanwhile, $\langle |{F^{\mathrm {H}}_{\mathrm {x, true}}}| \rangle$ and $\langle |{F^{\mathrm {H}}_{\mathrm {y, true}}}| \rangle$ strongly depend on *N*, whereas the absolute errors, $\langle |{F^{\mathrm {H}}_{\mathrm {x, true}} - F^{\mathrm {H}}_{\mathrm {x, pred}}}| \rangle$ and $\langle |{F^{\mathrm {H}}_{\mathrm {y, true}} - F^{\mathrm {H}}_{\mathrm {y, pred}}}| \rangle$, do not. This result suggests that the predicted velocity fields may have a certain degree of error that does not depend on the magnitude of the flow velocity. Given the dependence of the errors on *N*, the present deep-learning model will have good generalization for high cylinder numbers.

## Conclusion

This study constructed a deep-learning model that can predict a steady flow passing objects and addressed the prediction of steady fluid flow passing many fixed cylinders. The constructed deep-learning model is based on Hennigh’s model^20^ and outputs the *x*- and *y*-components of the flow-velocity field when the cylinder arrangement is input. The SPM was used to generate the training dataset^23, 25^. In the SPM, the shape and arrangement of objects (i.e., cylinders in the present study) are described by a smoothed profile function. The present deep-learning model uses values of the smoothed profile function as the input data.

Training and validation losses showed that the present model successfully learns the steady flow passing fixed cylinders. No overfitting was observed during the training. The present study investigated the accuracy of the predicted velocity field focusing on the velocity profiles of the fluid flow and the fluid force acting on the cylinders. The results show that the flow is successfully predicted by the present model when the number of cylinders is equal to or close to that set in the training dataset. The extrapolation of the prediction to a smaller number of cylinders results in error relating to internal friction of the fluid. The results of the fluid force acting on the cylinders also suggest that the present deep-learning model has good generalization performance for systems with a larger number of cylinders.

Here, the benefit of applying the deep-learning technique to the present problem should be explained in terms of computation time. In the present study, the computation time for the learning of the model is $T_{\mathrm {learn}} \sim {70}\,{\mathrm{h}}$. On the other hand, the computation time for the CFD simulation to obtain one example is $T_{\mathrm {sim}} \sim {4}\,{\mathrm{min}}$ with a single node of Intel$^{\textregistered }$ Xeon$^{\textregistered }$ CPU E5-2697A v4 processor. Judging from the computation times, there seems to be no benefit of applying the deep-learning technique to address the present problem. However, it is worth applying to the present problems considering its “scalability”. The deep-learning model can predict the flow in a very short period of inference time $T_{\mathrm {inf}} \sim {1}\,{\mathrm{s}}$ once it is trained. To obtain flows around solids of different shapes and arrangements using the model, it takes time $T_{\mathrm {inf}}$ since no re-training is required. On the other hand, typical CFD simulations require re-calculation, and it takes time $T_{\mathrm {sim}}$ to obtain the flow in the same situation. Therefore, the deep-learning model is useful to reduce the computation cost in the repeated calculation with changing shape and arrangement of objects, which is often required in the field of engineering. In this case, the more times the calculation is repeated, the more efficiently the calculation is done by the model.

The ultimate aim of the present research is to accelerate the CFD simulation of a fluid with solid objects using deep-learning techniques. The present deep-learning model has the potential to be used for the acceleration of the simulation of a multiphase flow because the model is able to predict the flow velocity field around many fixed cylinders with a very short inference time. The present model, however, takes only the positions and shapes of solid objects as the input, which is insufficient for replacing the fluid calculation of multiphase flow simulations. If the calculation in the SPM is to be replaced with a deep-learning model, the velocities of solid objects should be input into the model in addition to the positions and shapes of the solid objects. Following the present study, another deep-learning model that treats the velocities of solid objects by extending the present model was therefore constructed. The results will be presented in an adjoining paper.

## Supplementary Information

Supplementary Information.

## Data Availability

The data that support the findings of this study are available from the corresponding author upon reasonable request.
