# Supplementary material for: Prediction of steady flows passing fixed cylinders using deep learning
Source: Sci Rep. 2022 Jan 10;12:447. doi: 10.1038/s41598-021-03651-8 (PMC8748461; doi:10.1038/s41598-021-03651-8)
Supplement: Supplementary file 1 — Supplementary Information. [file 41598_2021_3651_MOESM1_ESM.pdf]

Supplementary Information for

Prediction of steady flows passing fixed cylinders using deep learning

Hiroto Ozaki[1,*] **and Takeshi Aoyagi**[1]

[1]Research Center for Computational Design of Advanced Functional Materials, National Institute of Advanced Industrial Science and Technology, Central 2, 1-1-1, Umezono, Tsukuba, Ibaraki, 305-8568, Japan
[*]h.ozaki@aist.go.jp

S1 Ablation study

As shown in Section 4, the present deep-learning model is constructed by making three modifications to Hennigh's model[1] in three respects: (1) Input data, (2) Degrees of freedom, and (3) Normalization technique. Here, the ablation study is carried out by removing each modification to test the effectiveness of the changes.

In this study, the following four models were compared. Model (a) is the present model shown in Section 4. Models (b)–(d) are the ones respectively constructed by removing each of the modifications (1)–(3) from Model (a) as follows. Model (b) takes the binarized smoothed profile function $\hat{\phi}$ instead of ϕ as input data. The binarized function $\hat{\phi}$ is defined as follows:

$$\hat{\phi} \equiv \begin{cases} 1 & (\phi \geq 0.5), \\ 0 & (\phi < 0.5). \end{cases} \tag{S1}$$

In Model (c), the number of down-sample and up-sample operations, the filter size, and the keep probability of the dropout layer are set the same as in Hennigh's model[1]. That is, the number of down-sample operations (which is the same as that of up-sample operations) is reduced from 5 to 4. The filter size f_i' of the ith residual block of Model (c) is set as $f_i' = f_i/3$ $(1 \leq i \leq 5)$ where f_i is the filter size of the ith residual block of Model (a) (see Figure 2). The keep probability of the dropout layer of the ith residual block, which is changed for each layer in Model (a), is set as constant value $k_i = 0.7$. In Model (d), the batch normalization layers before the activation function are disabled. These models are trained in the same manner as described in Section 4 and examined from the viewpoint of the train and validation losses.

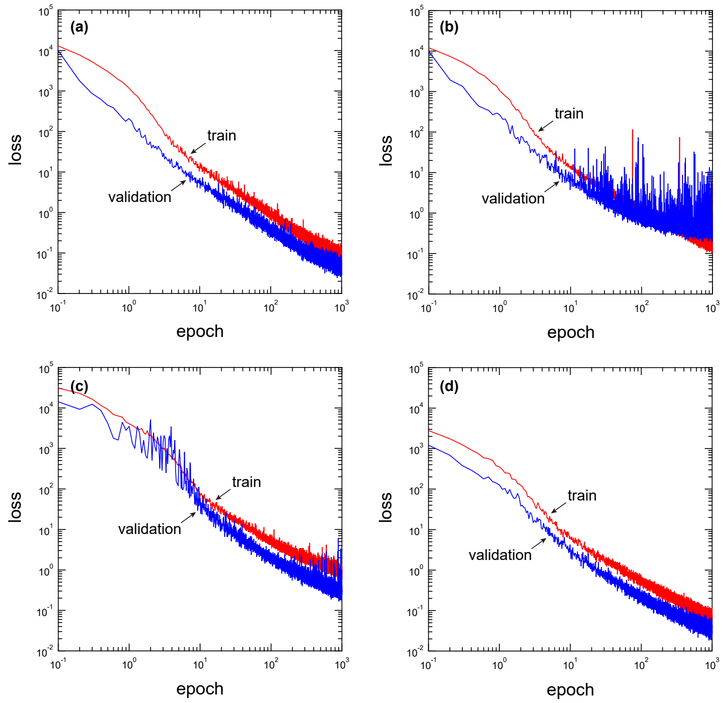

Figure S1. Training and validation losses per mini-batch as functions of the number of epochs (red: training loss, blue: validation loss). Figure (a)–(d) respectively show the results of Model (a)–(d).

Figure S1 shows the losses obtained by the four models. Figure S1 (a)–(d) respectively show the results of Model (a)–(d). From the results of Model (b) and (c), the effectiveness of modifications in (1) and (2) can be confirmed. From the results of Model (d), the effectiveness of the batch normalization layer cannot be confirmed for the flow treated in the present study. In the recent investigation, however, the batch-normalization technique is crucial for stable learning when the velocity of the center of gravity of cylinders is varied. (The result will be presented in the forthcoming paper.) The present paper leaves all three modifications for the generality of the model.

References

1. Hennigh, O. Steady state flow with neural nets. https://github.com/loliverhennigh/Steady-State-Flow-With-Neural-Nets.
